# Risk Factors for Pericallosal Artery Aneurysm Rupture Based on Morphological Computer-Assisted Semiautomated Measurement and Hemodynamic Analysis

**DOI:** 10.3389/fnins.2021.759806

**Published:** 2021-11-18

**Authors:** Xiaodong Zhai, Jiewen Geng, Chengcheng Zhu, Jiaxing Yu, Chuanjie Li, Nan Jiang, Sishi Xiang, Gang Fang, Peng Hu, Hongqi Zhang

**Affiliations:** ^1^Department of Neurosurgery, Xuanwu Hospital, Capital Medical University, Beijing, China; ^2^China International Neuroscience Institute, Beijing, China; ^3^Department of Radiology, University of Washington School of Medicine, Seattle, WA, United States; ^4^Department of Neurosurgery, Shunyi District Hospital, Beijing, China; ^5^Department of R&D, UnionStrong (Beijing) Technology Co., Ltd., Beijing, China

**Keywords:** intracranial aneurysm, pericallosal artery aneurysm, risk of rupture, morphology parameters, hemodynamic analysis

## Abstract

**Background:** Although pericallosal artery aneurysms (PAAs) are relatively uncommon, accounting for only 1–9% of all intracranial aneurysms (IAs), they exhibit a considerably high propensity to rupture. Nevertheless, our current knowledge of the risk factors for PAA rupture is still very limited. To fill this gap, we investigated rupture risk factors for PAAs based on morphological computer-assisted semiautomated measurement (CASAM) and hemodynamic analysis.

**Methods:** Patients with PAAs were selected from the IA database in our institute and their baseline data were collected. Morphological parameters were measured in all enrolled patients by applying CASAM. Computational fluid dynamics simulation (CFD) was performed to evaluate the hemodynamic difference between ruptured and unruptured PAAs.

**Results:** From June 2017 to June 2020, among 2141 patients with IAs in our institute, 47 had PAAs (2.2%). Thirty-one patients (mean age 57.65 ± 9.97 years) with 32 PAAs (20 unruptured and 12 ruptured) were included in the final analysis. Comparing with unruptured PAAs, ruptured PAAs had significantly higher aspect ratio (AR), mean normalized wall shear stress (NWSS), and mean oscillatory shear index (OSI) values than the unruptured PAAs (all *P* < 0.05) in univariate analyses. Multivariable analysis showed that a high mean OSI was an independent risk factor for PAA rupture (OR = 6.45, 95% CI 1.37–30.32, *P* = 0.018).

**Conclusion:** This preliminary study indicates that there are morphological and hemodynamic differences between ruptured and unruptured PAAs. In particular, a high mean OSI is an independent risk factor for PAA rupture. Further research with a larger sample size is warranted in the future.

## Introduction

Pericallosal artery aneurysms (PAAs), also known as distal anterior cerebral artery (DACA) aneurysms, are defined as intracranial aneurysms (IAs) located on the anterior cerebral artery (ACA) distal to the anterior communicating artery (Dinc et al., 2017; Petr et al., 2017). Aneurysm rupture lead to subarachnoid hemorrhage (SAH), a devastating condition with a high mortality rate of 30–40% (Morita et al., 2012; Li et al., 2013; Molyneux et al., 2015). Despite accounting for only a small percentage (1–9%) of all IAs, PAAs merit special attention due to their considerably high propensity to rupture (Lehecka et al., 2008b; Gross et al., 2014; Thompson et al., 2015; Korja et al., 2017). Multiple previous studies have reported that the odds ratio for a proportion of ruptured PAAs compared with IAs at all other locations is in the range of 2.5–4.7, indicating the higher rupture risk of PAAs (Weir et al., 2002; Bijlenga et al., 2013; Gross et al., 2014). Therefore, identifying high-rupture-risk PAAs and subsequently providing selective therapy are of critical importance to these patients.

Unfortunately, knowledge of the risk factors of PAAs for rupture remains limited due to the rarity of these lesions. Previous studies demonstrated that certain morphological parameters, e.g., aneurysm diameter, aspect ratio (AR), size ratio (SR), and irregular shape, are highly correlated with IA rupture (Ryu et al., 2011; Kashiwazaki et al., 2013; Greving et al., 2014; Wang et al., 2021). Degenerative remodeling of the artery wall caused by abnormal hemodynamics is believed to play an important role in the progression and rupture of IAs (Meng et al., 2007, 2014; Kulcsar et al., 2011; Tanaka et al., 2018). Thus, rupture risk evaluation based on morphological and hemodynamic parameters is critical for the identification of patients with PAAs at high risk for rupture.

We previously reported our initial experiences of morphological risk factor analysis for PAAs based on manual morphological measurements, which provided a solid basis for further exploration (Zhai et al., 2020). The present study goes a step further by providing a more detailed analysis of rupture risk factors for PAAs based on more advanced morphological computer-assisted semiautomated measurement (CASAM) and hemodynamic analysis.

## Materials and Methods

### Patient Selection

We retrospectively reviewed our prospectively collected database of 2141 consecutive patients with IAs who underwent microsurgery or interventional therapy between June 2017 and June 2020. Traumatic, dissecting, and fusiform aneurysms were excluded prior to the initial screening. Of those patients, 47 had PAAs, accounting for 2.20% of the 2141 patients with IAs. Of the 47 patients with PAAs, 16 had two-dimensional (2D) digital subtraction angiography (DSA) data only, which did not fulfill the requirements of CASAM and hemodynamic analysis. Therefore, the present analysis included the remaining 31 patients with 32 PAAs (20 unruptured and 12 ruptured) who underwent three-dimensional (3D) DSA in the final analysis. Baseline and clinical patient data were collected from the medical records and the aneurysm database.

### Computer-Assisted Semiautomated Measurement

**FIGURE 1:**
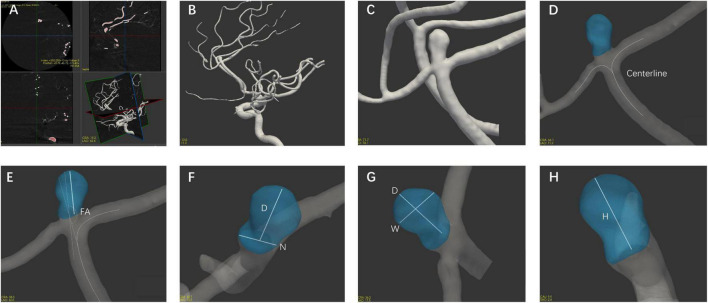
Flow chart of the CASAM method for measuring the morphological parameters of aneurysms. **(A)** Import of original data; **(B)** 3D vessel reconstruction; **(C)** selection of region of interest; **(D)** segmentation of aneurysm using centerline method; and **(E–H)** measurement of morphological parameters of aneurysm and export of all parameters. D, diameter; N, neck width; W, width; H, height; FA, flow angle; PA, parent artery diameter.

**FIGURE 2:**
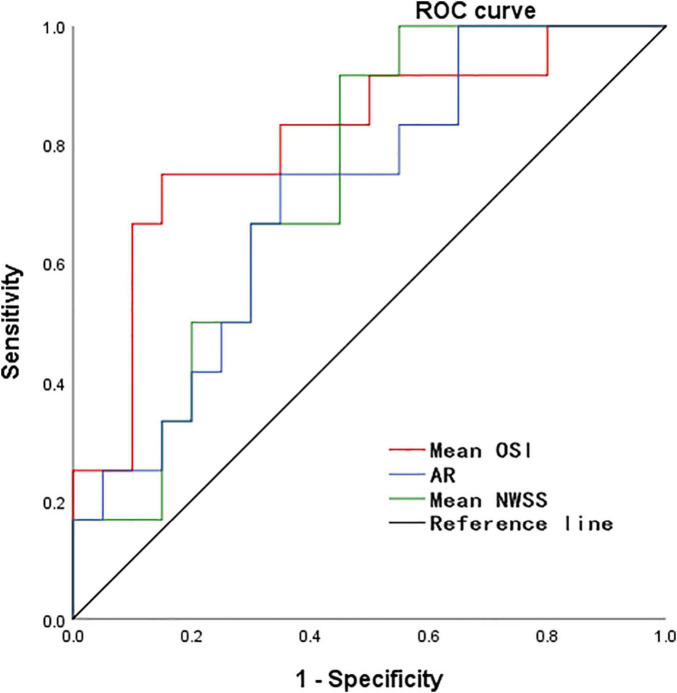
The ROC curve for predicting PAA rupture. ROC, receiver operating characteristic, NWSS, normalized wall shear stress; OSI, oscillatory shear index; AR, aspect ratio.

**FIGURE 3:**
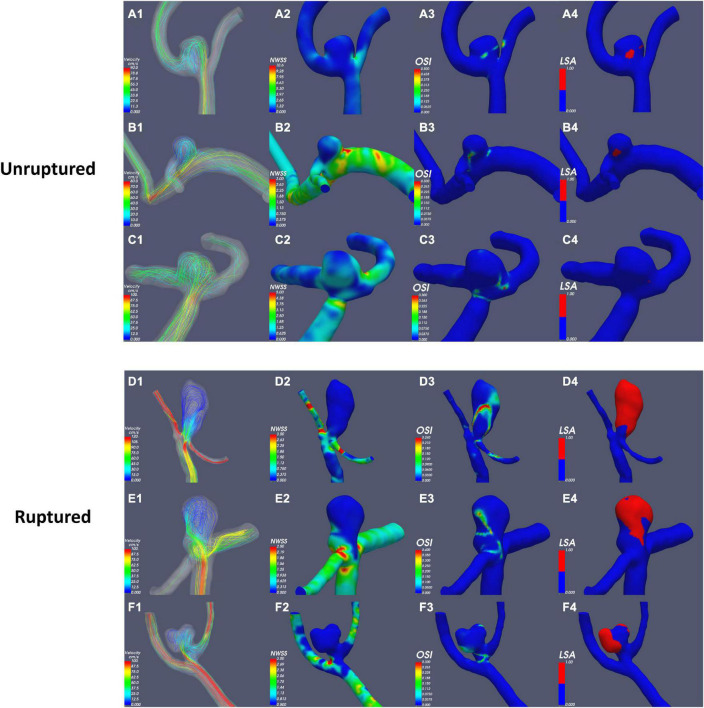
Comparison of hemodynamic parameters between typical ruptured and unruptured PAAs. NWSS, normalized wall shear stress; OSI, oscillatory shear index; LSA, low-shear area.

The morphological parameters were measured in all enrolled patients with 3D DSA data using CASAM, the methodology for which was presented in detail in our previous report (Geng et al., 2020). As shown in Figure 1, the main steps followed in the CASAM procedure are as follows: (1) importing of original DSA tomographic data (Figure 1A); (2) 3D blood vessel reconstruction (Figure 1B); (3) manual selection of the region of interest (ROI) of the vessel where the aneurysm is located (Figure 1C); (4) automated extraction of the centerline and segmentation of the aneurysm (Figure 1D); and (5) computer-assisted automated measurement of 3D morphological parameters and exportation of all parameters (Figures 1E–H). CASAM enabled us to acquire the following morphological and derived parameters: (1) diameter (D): the maximum distance from the center of aneurysm neck to a point on the sac; (2) neck width (N): the maximum diameter in the neck plane; (3) width (W): the maximum distance between two points in the aneurysm sac perpendicular to the diameter of the aneurysm; (4) height (H): the maximum distance from the plane of the neck to the surface of the aneurysm; (5) flow angle (FA): the angle between the direction of blood flow and the diameter of the aneurysm; (6) parent artery diameter (PA): the mean diameter of parent artery between 3.0 cm upstream of the neck and 3.0 cm downstream; (7) AR: the ratio of the diameter of the aneurysm to the width of the aneurysm neck, AR = D/N; (8) SR: the ratio of the diameter of the aneurysm to the diameter of the parent artery, SR = D/PA; and (9) D/W: the ratio of the diameter to the width of the aneurysm. All CASAM procedures were completed by one experienced neurointerventionist due to the perfect interclass consistency of CASAM. Aneurysms with irregular shape, defined as having lobular or daughter sacs, was identified by two experienced neuroradiologists according to a previous study (Lindgren et al., 2016). If there was any disagreement, it was resolved by a third reader with 25 years of experience in neurovascular imaging.

### Image Reconstruction and Computational Fluid Dynamics Modeling

For this study, pre-treatment patient-specific 3D-DSA data of all PAAs were obtained. The methods for image reconstruction and computational fluid dynamics simulation (CFD) simulation of hemodynamic studies were detailed in our previous publications (Hu et al., 2015a,b; Qin et al., 2017). Firstly, 3D models were generated with Raw DICOM format images and processed using Mimics medical software (Version 19.0, Materialise, Leuven, Belgium) and Geomagic Studio version 12.0 (Geomagic Inc., Cary, NC, United States), saved as standard tessellation language format as the input for the next step (Qin et al., 2017; Liu et al., 2021).

Each patient-specific 3D model was subdivided into the aneurysm sac and parent artery regions before meshing. Parent artery was defined as a 20-mm long segment of vessel surrounding the aneurysm sac. Then, the geometry of each 3D model was imported into ICEM CFD software (ANSYS Inc., Canonsburg, PA, United States). Different mesh sizes were set for different parts. For the inlet, outlet, and sac, we chose a mesh size of 0.1 mm, which is appropriate for aneurysms in the typical size range; for the parent artery, we used a mesh size of 0.3 mm.

The total number of finite-volume tetrahedral grid elements was approximately 1 million, and four layers of prism elements were used for the CFD simulations. The elements number was set based on grid-independence analysis to obtain high quality simulation results. The Navier–Stokes equations were the governing equations employed in the current study.

After meshing, ANSYS CFX 18.0 (ANSYS Inc.) was used for hemodynamic simulation. For the model, blood was assumed to be a Newtonian fluid with a density of 1060 kg/m^3^ and a dynamic viscosity of 0.0035 N s/m^2^. The vessel wall was assumed to be rigid with a no-slip boundary. Because the patient-specific boundary conditions were not available, the inflow boundary condition was a representative pulsatile velocity profile obtained from the average normal human (Ford et al., 2005). A traction-free boundary condition was applied to all outlets (Cebral et al., 2005). Initial pressure and velocity were set to zero. Three cardiac cycles were simulated to minimize transient numerical errors. The results from the third simulated cardiac cycle were collected as output for the final analyses. Validation of these methods (consistency, reliability) has been demonstrated in our previous publication (Hu et al., 2015a,b). A flowchart demonstrating an outline of the image reconstruction and CFD procedures is included in Supplementary Figure 1.

### Hemodynamic Analysis

Several common and important hemodynamic parameters were quantified and used to characterize the hemodynamics of IAs. Normalized wall shear stress (NWSS) and normalized pressure (NP), defined as the WSS and pressure of the aneurysm wall divided by that of the parent artery wall, respectively, were calculated to allow comparisons among different patients (Jou et al., 2008; Yuan et al., 2020). The wall shear stress gradient (WSSG) was calculated based on the simulated pulsatile flow simulations (Hu et al., 2015a,b). The oscillatory shear index (OSI) is a non-dimensional parameter that is defined as the directional change in WSS during the cardiac cycle (Soldozy et al., 2019). The combined hemodynamic parameter (CHP) can be defined as a weighted average of the WSS and OSI so that each hemodynamic parameter contributes proportionately to the final CHP (Cho et al., 2018). The relative residence time (RRT) is a marker of disturbed blood flow, that incorporates particle variability during the cardiac cycle (Riccardello et al., 2018). Ultimately, hemodynamic parameters, including NWSS, NP, WSSG, CHP, RRT, and OSI, were calculated to evaluate the hemodynamic difference between the ruptured and unruptured PAAs. To ensure repeatability, all measurements were conducted at least twice.

### Statistical Methods

Continuous variables were presented as the mean ± SD, and categorial variables were presented as absolute numbers with percentages. Data were tested for normality by constructing P–P and Q–Q plots. Categorical variables were compared between groups using the Pearson χ^2^ test, continuity correction, and a two-tailed Fisher’s exact test. Continuous variables were compared between groups using Student’s *t*-test. We also evaluated the predictive value of factors that were statistically significant in the univariate analysis by receiver operating characteristic (ROC) analysis. A multivariable binary logistic regression analysis was performed including significant factors in univariate analysis. Statistical significance was defined as *P* < 0.05. Statistical analysis was performed using SPSS Statistics version 24.0 software (IBM Corp., Armonk, NY, United States).

## Results

The mean age of the patients in the PAA cohort was 57.65 ± 9.97 years (range: 33–84 years). A total of 59.4% (19/32) of the PAAs were located at the bifurcation of the callosomarginal artery in the A3 segment, and no significant difference in baseline and morphological characteristics was detected between the ruptured and unruptured groups. Overall, there was little significant difference in the baseline and morphologic characteristics between ruptured and unruptured PAAs, and only AR was significantly higher in the ruptured group than in the unruptured group (*P* = 0.045) (Table 1).

**TABLE 1:** Baseline and morphological characteristics of the included patients and aneurysms.

**Variable**		**Unruptured (*n* = 19 Patients; *n* = 20 aneurysms)**	**Ruptured (*n* = 12 Patients; *n* = 12 aneurysms)**	***P*-value**
Age		57.37 ± 5.6	58.08 ± 14.81	0.875
Gender	Male	5 (26.3%)	5 (41.7%)	0.620
	Female	14 (73.7%)	7 (58.3%)	
Hypertension		13 (68.4%)	10 (83.3%)	0.433
Heart disease		1 (5.3%)	2 (16.7%)	0.543
Irregular shape		6 (30.0%)	7 (58.3%)	0.227
Location	Side wall	4 (20.0%)	2 (16.7%)	0.99
	Bifurcation	16 (80.0%)	10 (83.3%)	
Side	Left	9 (45.0%)	7 (58.3%)	0.716
	Right	11 (55.0%)	5 (41.7%)	
Multiple aneurysms		11 (55.0%)	6 (50.0%)	0.990
Located in anterior A3		12 (60.0%)	7 (58.3%)	0.99
Diameter (mm)		3.38 ± 2.18	3.77 ± 1.98	0.619
Width (mm)		3.77 ± 1.68	3.6 ± 1.33	0.771
Height (mm)		2.84 ± 1.51	2.96 ± 1.78	0.843
Neck (mm)		4.14 ± 2.38	3.26 ± 0.78	0.223
Flow angle (°)		126.07 ± 24.26	121.34 ± 40.55	0.681
PA		1.93 ± 0.44	1.65 ± 0.48	0.105
SR		1.87 ± 1.28	2.43 ± 1.51	0.272
AR		0.82 ± 0.33	1.15 ± 0.57	0.045
D/W		0.87 ± 0.33	1.04 ± 0.32	0.181

*As defined in previous studies, the anterior A3 site is located at the bifurcation of the callosomarginal artery in the A3 segment (Lehecka et al., 2008a,b). PA, parent artery diameter; AR, aspect ratio; SR, size ratio; D/W, diameter/width.*

According to the results of the quantitative hemodynamic analysis shown in Table 2, the mean OSI was significantly higher in the ruptured group than in the unruptured group (*P* = 0.035), and the mean NWSS was significantly lower in the ruptured group than in the unruptured group (*P* = 0.012). ROC curve analysis was performed to evaluate the predictive value of the variables for the rupture of PAAs (Figure 2). The largest AUC was that of the mean OSI (AUC = 0.808), and the AUC of mean NWSS (AUC = 0.733) ranked second, followed by the AUC of the AR (AUC = 0.712). The optimal threshold for mean OSI was 0.009875 with 75.0% sensitivity and 85.0% specificity. The optimal threshold for the mean NWSS was 0.78, which provided 91.7% sensitivity and 55.0% specificity. The optimal threshold for AR was 0.82, with a sensitivity of 75% and specificity of 65%. Factors in univariate analysis with *P* < 0.05 were subsequently used in a multivariable binary logistic regression analysis, including AR, mean NWSS, and mean OSI. The results of the multivariate analysis indicated that a high mean OSI was significantly associated with PAA rupture (OR = 6.45, 95% CI 1.37–30.32, *P* = 0.018). Multiple vital hemodynamic parameters are compared between several typical ruptured and unruptured typical PAAs in Figure 3.

**TABLE 2:** Hemodynamic parameters of the ruptured and unruptured PAA groups.

**Variable**	**Unruptured (*n* = 20 aneurysms)**	**Ruptured (*n* = 12 aneurysms)**	***P*-value**
Maximum CHP	0.71050.1457	0.75740.1274	0.364
Minimum CHP	0.00250.0025	0.00210.0017	0.677
Mean CHP	0.13760.0863	0.17020.0805	0.298
LSA ratio	0.15220.2503	0.30090.3508	0.172
Maximum NP	1.7680.595	1.72050.4972	0.818
Minimum NP	0.79740.182	0.89740.3714	0.314
Mean NP	1.24190.2256	1.29680.1703	0.474
Maximum NWSS	4.23342.6437	3.37751.9244	0.338
Minimum NWSS	0.08740.1233	0.03540.0469	0.102
Mean NWSS	0.84270.5945	0.42210.2955	0.012
Maximum OSI	0.25180.122	0.3090.0927	0.173
Minimum OSI	0.00020.0001	0.00020.0001	0.981
Mean OSI	0.00820.0046	0.01860.0148	0.035
Maximum RRT	6.836112.3628	23.521851.7724	0.294
Minimum RRT	0.00680.0052	0.00920.0143	0.587
Mean RRT	0.25720.6068	0.47911.0488	0.452
Maximum WSSG	5772.49655047.1299	7295.35024775.8222	0.406
Minimum WSSG	10.651714.4013	5.02996.1093	0.211
Mean WSSG	525.0274353.7077	559.3032620.3051	0.843

*NWSS, normalized wall shear stress; NP, normalized pressure; OSI, oscillatory shear index; WSSG, wall shear stress gradient; LSA, low-shear area; CHP, combined hemodynamic parameter; RRT, relative residence time.*

## Discussion

To the best of our knowledge, this is the first study that assess hemodynamics and morphology simultaneously to evaluate the rupture risk of PAAs. In the present study, the results of the univariate analysis revealed that ruptured PAAs had significantly higher AR, mean NWSS, and mean OSI values than unruptured PAAs. AR represents the diameter-to-neck ratio, high AR of ruptured PAAs means that they have a relatively small neck area that limits the blood flow in the aneurysm sac and induces relatively slow and inconsistent flow in the aneurysm sac (Nader-Sepahi et al., 2004; Tateshima et al., 2010). As AR increases, vortices gradually appear in the aneurysm sac, and the flow velocity decreases (Ujiie et al., 1999; Tykocki et al., 2014). Most previous studies have indicated that AR is positively correlated with the risk of aneurysm rupture, consistent with our results (Ryu et al., 2011; Soldozy et al., 2019).

WSS is the frictional force induced by blood flow acting on the endothelium of the vessel wall, whose direction is parallel to local blood flow (Soldozy et al., 2019). For comparisons among different patients, NWSS was calculated for the hemodynamic analysis; this variable is defined as the WSS of the aneurysm wall divided by that of the parent artery wall. Low WSS was usually considered to be associated with aneurysm rupture (Miura et al., 2013; Soldozy et al., 2019), consistent with our finding that ruptured PAAs had significantly lower NWSS. OSI is a non-dimensional parameter that is defined as the directional change in WSS during the cardiac cycle; this frequency-based parameter has been used to quantify IA rupture (Soldozy et al., 2019). Our results indicated that a high mean OSI was an independent risk factor for PAA rupture (OR = 6.45, 95% CI 1.37–30.32, *P* = 0.018). OSI reflects the temporal variation in the blood flow direction, and the temporal variations in blood flow can be illustrated by the continuously emerging and disappearing vortices of blood flow throughout the cardiac cycle (Can and Du, 2016; Soldozy et al., 2019). OSI reflects the shape of the aneurysm and its parent artery because aneurysmal geometry and hemodynamics are intertwined, consistent with our finding (see above) that ruptured PAAs tend to have a relatively elongated shape as well as inconsistent flow.

Despite comprehensive screen, in an aneurysm cohort of 2141 patients, only 2.20% of them had PAAs. The primary limitation of this study was the small sample size, which might limit its generalizability. Second, the 3D DSA examination used for the hemodynamic study was not administered to all patients with PAAs, which caused a certain degree of selective bias. To overcome these limitations, further analysis with more adequate data is warranted in the future.

## Conclusion

This study suggested that there were morphological and hemodynamic differences between ruptured and unruptured PAAs. In particular, a high mean OSI is an independent risk factor for PAA rupture. These findings should be considered preliminary until confirmed in larger studies.

## Data Availability Statement

The original contributions presented in the study are included in the article/Supplementary Material, further inquiries can be directed to the corresponding authors.

## Ethics Statement

The studies involving human participants were reviewed and approved by the Ethics Committee of Xuanwu Hospital. The patients/participants provided their written informed consent to participate in this study. Written informed consent was obtained from the individual(s) for the publication of any potentially identifiable images or data included in this article.

## Author Contributions

XZ, JG, CZ, CL, JY, NJ, SX, GF, PH, and HZ contributed to the conception, design, analysis, and interpretation of the data as well as to drafting the manuscript and revising it critically. All authors have read and approved the final version of the manuscript.

## Conflict of Interest

GF was employed by the company UnionStrong (Beijing) Technology Co., Ltd. The remaining authors declare that the research was conducted in the absence of any commercial or financial relationships that could be construed as a potential conflict of interest.

## Publisher’s Note

All claims expressed in this article are solely those of the authors and do not necessarily represent those of their affiliated organizations, or those of the publisher, the editors and the reviewers. Any product that may be evaluated in this article, or claim that may be made by its manufacturer, is not guaranteed or endorsed by the publisher.

## Supplementary Material

Supplementary Figure 1 Flowchart of image reconstruction and CFD procedures based on patient-specific angiogram.
