# Supplementary figures and images for: Risk Factors for Pericallosal Artery Aneurysm Rupture Based on Morphological Computer-Assisted Semiautomated Measurement and Hemodynamic Analysis
Source: Front Neurosci. 2021 Nov 18;15:759806. doi: 10.3389/fnins.2021.759806 (PMC8636593; doi:10.3389/fnins.2021.759806)

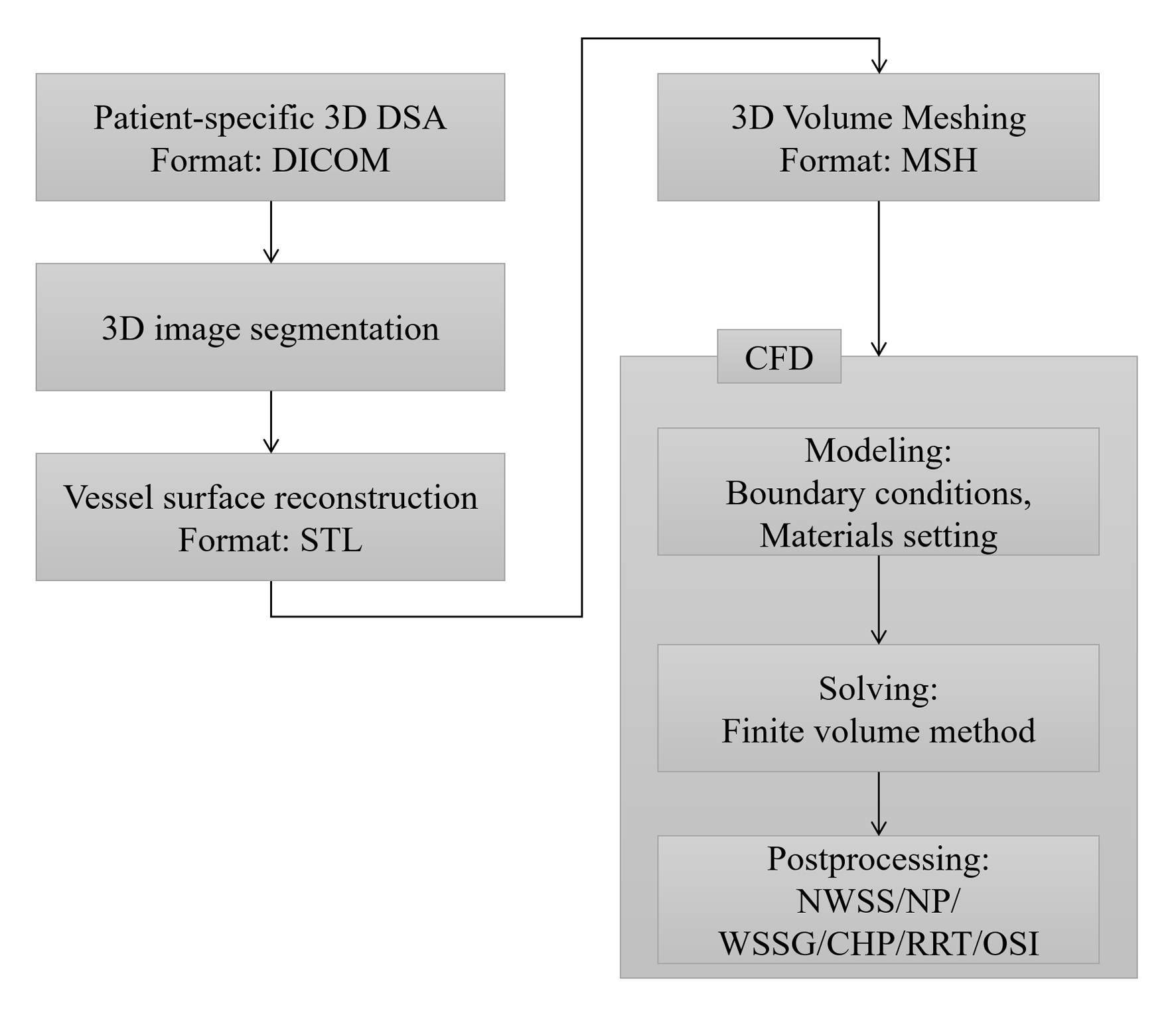

Supplement: Supplementary Figure 1 — Flowchart of image reconstruction and CFD procedures based on patient-specific angiogram. [file Image_1.TIF]
